# Pre-existing neutralizing antibodies prevent CD8 T cell-mediated immunopathology following respiratory syncytial virus infection

**DOI:** 10.1038/s41385-019-0243-4

**Published:** 2019-12-16

**Authors:** Megan E. Schmidt, David K. Meyerholz, Steven M. Varga

**Affiliations:** 1 Interdisciplinary Graduate Program in Immunology, University of Iowa, Iowa City, IA 52242 USA; 2 Department of Pathology, University of Iowa, Iowa City, IA 52242 USA; 3 Department of Microbiology and Immunology, University of Iowa, Iowa City, IA 52242 USA

## Abstract

Despite being a leading cause of severe respiratory disease, there remains no licensed respiratory syncytial virus (RSV) vaccine. Neutralizing antibodies reduce the severity of RSV-associated disease, but are not sufficient for preventing reinfection. In contrast, the role of memory CD8 T cells in protecting against a secondary RSV infection is less established. We recently demonstrated that high-magnitude memory CD8 T cells efficiently reduced lung viral titers following RSV infection, but induced fatal immunopathology that was mediated by IFN-γ. To evaluate the ability of RSV-specific neutralizing antibodies to prevent memory CD8 T cell-mediated immunopathology, mice with high-magnitude memory CD8 T cell responses were treated with neutralizing antibodies prior to RSV challenge. Neutralizing antibody treatment significantly reduced morbidity and prevented mortality following RSV challenge compared with IgG-treated controls. Neutralizing antibody treatment restricted early virus replication, which caused a substantial reduction in memory CD8 T cell activation and IFN-γ production, directly resulting in survival. In contrast, therapeutic neutralizing antibody administration did not impact morbidity, mortality, or IFN-γ levels, despite significantly reducing lung viral titers. Therefore, only pre-existing neutralizing antibodies prevent memory CD8 T cell-mediated immunopathology following RSV infection. Overall, our results have important implications for the development of future RSV vaccines.

## Introduction

Respiratory syncytial virus (RSV) is a common cause of severe respiratory disease in young children, the elderly, and immunocompromised populations.^1–5^ RSV causes an estimated 33 million acute lower respiratory tract infections annually in children under 5 years of age, with over three million episodes requiring hospitalization.^6^ Despite the immense healthcare burden attributed to RSV infection, there is currently no licensed RSV vaccine. A lack of complete understanding of the correlates of immunity against RSV has remained a major impediment in RSV vaccine development. Neutralizing antibodies have been shown to reduce the severity of RSV-associated disease and thus remain an important goal of RSV vaccination.^7–10^ However, high levels of neutralizing antibodies are not able to prevent RSV-induced disease in all individuals.^11,12^ In addition, high neutralizing antibody titers alone are insufficient to prevent reinfection with RSV in infants, young children, and adults.^9,11,13–15^ Therefore, RSV vaccines in which neutralizing antibodies are the sole immune mediator for preventing secondary infection will likely not provide long-term protection in all individuals.

In contrast to neutralizing antibodies, the protective capacity of cell-mediated immunity against RSV infection has received substantially less attention. It has been well established that CD8 T cells provide protection during an acute RSV infection by mediating viral clearance.^16–21^ Thus, memory CD8 T cells may also provide protection against reinfection with RSV and may be desirable to elicit through vaccination. We recently evaluated the capacity for RSV-specific memory CD8 T cells to provide protection against secondary RSV infection in the absence of RSV-specific memory CD4 T cells and antibodies.^22^ Utilizing a dendritic cell—*Listeria monocytogenes* (DC-LM) prime-boost immunization approach, high-magnitude, systemic memory CD8 T cells specific to the M2_82–90_ (M2_82_) immunodominant CD8 T cell epitope were generated in BALB/c mice. M2_82_-immunized mice exhibited a significant reduction in lung viral titers following RSV challenge compared with controls undergoing an acute RSV infection. However, despite enhanced viral clearance, M2_82_-immunized mice unexpectedly exhibited enhanced weight loss, pulmonary dysfunction, and mortality. The exacerbated morbidity and mortality observed in M2_82_-immunized mice was mediated by the rapid IFN-γ production by memory CD8 T cells in the lung and airways.^22^ These results indicate that CD8 T cell epitope-specific vaccinations could have detrimental consequences following a subsequent, natural RSV infection in humans. However, given the established capacity of neutralizing antibodies to prevent RSV-associated disease severity, it is likely that the combined induction of both memory CD8 T cells and neutralizing antibodies could be an efficacious vaccine strategy.

Herein, we evaluated the ability of RSV-specific neutralizing antibodies to prevent memory CD8 T cell-mediated immunopathology following RSV infection. M2_82_-immunized mice prophylactically treated with the RSV-specific neutralizing antibodies motavizumab or D25 exhibited significantly ameliorated weight loss, pulmonary dysfunction, and mortality following RSV infection compared with treatment with a non-neutralizing RSV-specific antibody or control IgG. Prophylactic motavizumab and D25 treatment restricted early virus replication, resulting in significantly reduced lung and serum IFN-γ levels, CD8 T cell activation, and memory CD8 T cell IFN-γ production in the lung and airways ultimately leading to survival of the mice. In contrast, therapeutic administration of either motavizumab or D25 1 day after RSV infection significantly reduced viral titers, but failed to significantly impact morbidity, mortality, or lung and serum IFN-γ levels in M2_82_-immunized mice. These results suggest that prophylactic treatment with RSV-specific neutralizing antibodies prevent memory CD8 T cell-mediated immunopathology following RSV infection, whereas treatment after RSV challenge is not sufficient to do so. Our results demonstrate that pre-existing neutralizing antibodies effectively protect against CD8 T cell-mediated disease, indicating that the induction of both RSV-specific memory CD8 T cells and neutralizing antibodies in combination may provide a successful vaccination strategy. Overall, our findings have important implications for RSV vaccine design and the prevention of RSV vaccine-enhanced disease.

## Results

### Prophylactic neutralizing antibody treatment provides protection against memory CD8 T cell-mediated immunopathology

**Fig. 1:**
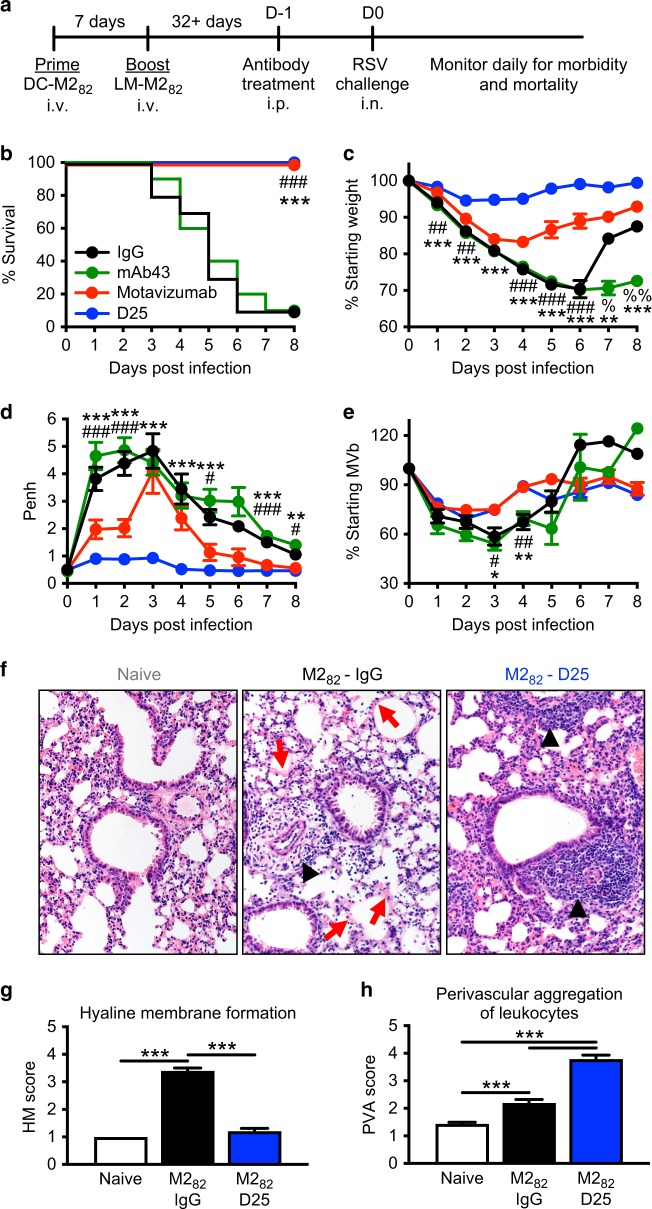
Prophylactic neutralizing antibody treatment prevents memory CD8 T cell-mediated morbidity and mortality. **a** Schematic depicting immunization protocol and antibody administration. Naive BALB/c mice were primed i.v. with mature DCs coated in M2_82_ peptide and boosted i.v. 7 days later with LM-M2_82_. M2_82_-immunized mice were treated with IgG, mAb43, motavizumab, or D25 antibodies i.p. 32–40 days post boost. One day after antibody treatment, mice were challenged with RSV i.n. and monitored daily for (**b**) survival, (**c**) weight loss, and respiratory parameters, including (**d**) enhanced pause (Penh) and (**e**) minute volume (MVb). **f** Lungs were harvested on day 4 p.i. from M2_82_-immunized mice treated with either IgG or D25 and naive mice. Sections were stained for H&E, and representative images were captured at ×200 magnification. Red arrows highlight the formation of hyaline membranes (HM). Black arrowheads indicate perivascular aggregation of leukocytes (PVA). Data are presented as mean ± SEM of two independent experiments (*n* = 10 in (**b**–**e**); *n* = 5–8 in (**f**–**h**)). Groups were compared using a two-way ANOVA for (**b**–**e**) and one-way ANOVA in (**g**, **h**). ^*/#/%^*p* < 0.05, ^**/##/%%^*p* *<* 0.01, ^***/###/%%%^*p* < 0.001. Percent symbols represent statistical significance between IgG and mAb43 groups, pound signs represent statistical significance between IgG and motavizumab groups, and asterisks represent statistical significance between IgG and D25 groups.

Naive BALB/c mice were immunized with DCs pulsed with M2_82_ peptide and boosted 7 days later with recombinant LM expressing the M2_82_ CD8 T cell epitope to generate high-magnitude, systemic M2_82_-specific CD8 T cell responses as demonstrated previously (Fig. 1a).^22^ To determine the ability of pre-existing neutralizing antibodies to prevent CD8 T cell-mediated immunopathology following RSV infection, M2_82_-immunized mice were administered either motavizumab or D25 antibodies intraperitoneally (i.p.) 1 day prior to RSV challenge (Fig. 1a). Motavizumab, the next generation of the palivizumab monoclonal antibody used clinically, exhibits high in vitro neutralizing activity.^23^ Motavizumab is a potent binder of the RSV fusion (F) protein’s antigenic site II that is accessible on both the pre-F and post-F confirmations.^23^ D25 binds the highly antigenic site ∅ on the RSV F protein’s pre-F confirmation and displays highly potent in vitro neutralizing activity.^24,25^ In addition to motavizumab and D25 neutralizing antibodies, monoclonal antibody #43 (mAb43) was selected as a control antibody that binds to RSV F with high affinity, but lacks neutralizing activity in vitro.^26^ All groups were directly compared to mice receiving a rat IgG control antibody.

As previously demonstrated, M2_82_-immunized mice treated with control IgG exhibited substantial mortality following RSV challenge, with up to 90% of mice succumbing to infection (Fig. 1b).^22^ Approximately 60% of fatalities in IgG-treated mice were due to mice naturally succumbing to RSV infection, while 40% were euthanized upon reaching a humane weight loss endpoint. Similarly, mAb43 administration did not impact mortality, as mAb43-treated mice exhibited a nearly identical survival curve as control IgG-treated mice (Fig. 1b). Remarkably, M2_82_-immunized mice administered either motavizumab or D25 were completely protected against mortality, exhibiting 100% survival following RSV infection (Fig. 1b). In addition, both motavizumab- and D25-treated mice exhibited significantly reduced clinical disease manifestations, including weight loss and pulmonary dysfunction, compared with IgG controls (Fig. 1c–e). Interestingly, D25 administration reduced weight loss and enhanced pause (Penh) to a greater degree than motavizumab treatment, correlating with the potency of their in vitro neutralizing activity (Fig. 1c, d).^25^ In contrast, mAb43-treated mice exhibited weight loss and parameters of pulmonary dysfunction similar to IgG control-treated mice (Fig. 1c–e). Consistent with these results, neutralizing antibody treatment also provided protection against lung pathology as evaluated by histology. IgG-treated M2_82_-immunized mice exhibited extensive hyaline membrane formation (HM), scattered cellular debris, and leukocytes in airspaces, consistent with diffuse alveolar damage (Fig. 1f, g; Supplementary Fig. 1).^27,28^ In contrast, D25-treated mice showed rare evidence of HM but extensive perivascular aggregation of leukocytes (PVA), an indicator of acute lung inflammation, compared to IgG treatment (Fig. 1f–h; Supplementary Fig. 1). Thus, while D25-treated mice demonstrate acute lung inflammation, their lungs are significantly protected against the more severe lung pathology associated with diffuse alveolar damage observed in IgG-treated mice. Overall, these results suggest that pre-existing neutralizing antibodies provide protection against memory CD8 T cell-mediated weight loss, pulmonary dysfunction, and severe lung pathology and completely prevent mortality following RSV infection.

**Fig. 2:**
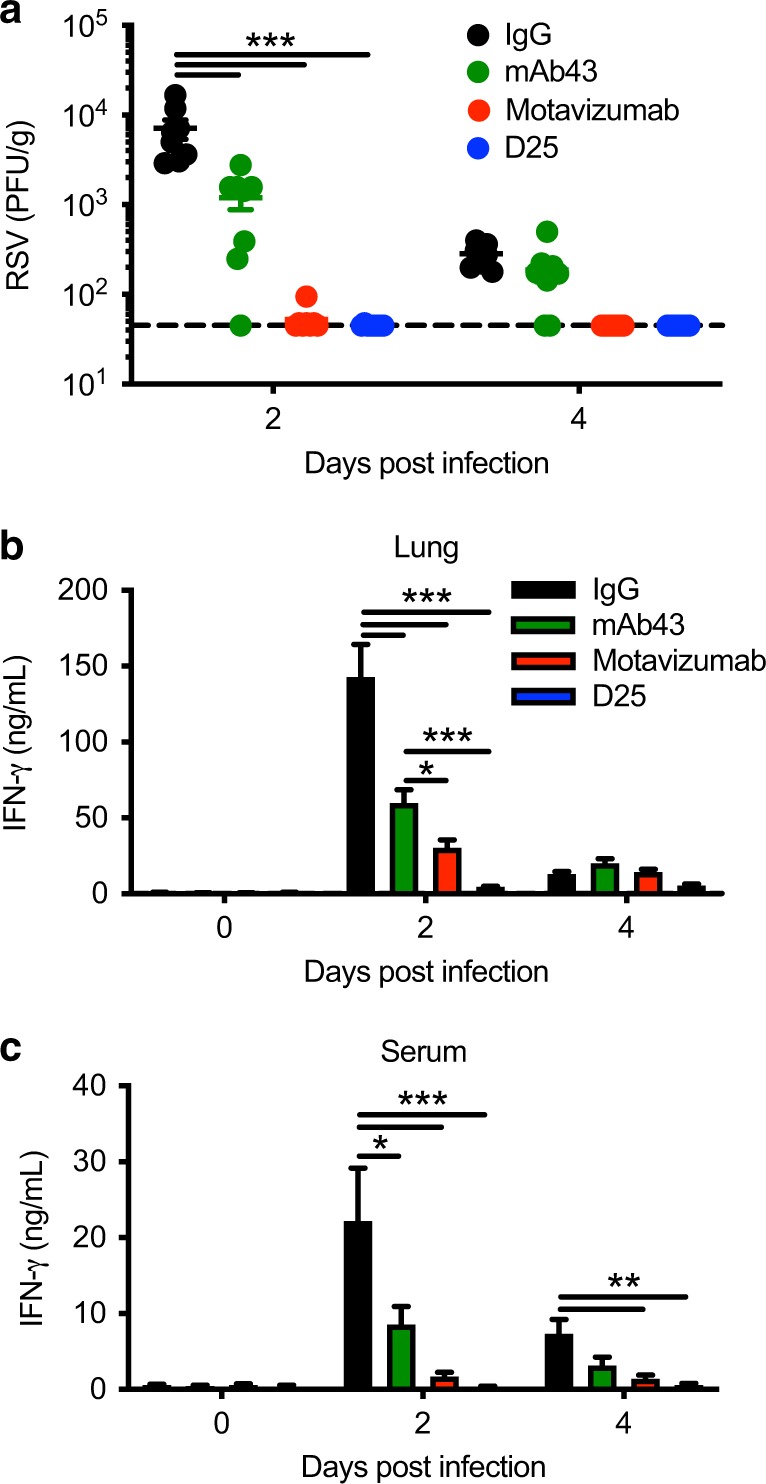
Prophylactic neutralizing antibody treatment restricts virus replication and significantly reduces IFN-γ levels following RSV challenge. M2_82_-immunized mice were treated with IgG, mAb43, motavizumab, or D25 antibodies i.p. and challenged with RSV i.n. 1 day later. **a** Lungs were harvested on days 2 and 4 p.i., and plaque assays were performed to determine RSV titers. Dashed lines indicate the limit of detection of the assay. **b**, **c** Lungs were harvested on days 0, 2, and 4 p.i., and IFN-γ protein levels in the (**b**) lung and (**c**) serum were determined by ELISA. Data are presented as mean ± SEM of two independent experiments (*n* = 8). Groups were compared using a two-way ANOVA. *p < 0.05, **p < 0.01, ***p < 0.001.

We previously demonstrated that RSV-specific memory CD8 T cells efficiently reduce lung viral titers following a secondary RSV infection in M2_82_-immunized mice.^22^ To determine whether RSV-specific neutralizing antibodies contribute to early viral clearance in M2_82_-immunized mice, we evaluated lung viral titers on days 2 and 4 post infection (p.i.) by plaque assay. Control IgG-treated M2_82_-immunized mice exhibited ~10^4^ plaque-forming units (PFU) in the lungs on day 2 p.i., which declined ~1.5 logs to a low level of detectable virus on day 4 p.i. (Fig. 2a). Interestingly, mAb43-treated mice displayed a significant decrease in lung viral titers compared with IgG controls on day 2 p.i., despite its lack of in vitro neutralizing activity (Fig. 2a). As expected, prophylactic treatment with either motavizumab or D25 restricted early virus replication, with only a single mouse in each group exhibiting detectable virus in the lung on day 2 p.i. and no virus detected on day 4 p.i. (Fig. 2a). These results indicate that the pre-existing neutralizing antibodies motavizumab and D25 restrict early virus replication in the lung of M2_82_-immunized mice.

IFN-γ levels were significantly elevated in the lung and serum of M2_82_-immunized mice compared with controls, and neutralization of IFN-γ completely protected against CD8 T cell-mediated mortality.^22^ Therefore, IFN-γ mediated the exacerbated morbidity and mortality observed following RSV infection in M2_82_-immunized mice. Because prophylactic neutralizing antibody treatment protected M2_82_-immunized mice from severe immunopathology, we next evaluated whether neutralizing antibody administration altered IFN-γ levels. As expected, IgG-treated M2_82_-immunized mice exhibited robust IFN-γ protein levels on day 2 p.i. in the lung (Fig. 2b). In contrast, treatment with either motavizumab or D25 resulted in significantly reduced lung IFN-γ levels on day 2 p.i., with D25 treatment inducing very little detectable IFN-γ (Fig. 2b). Interestingly, mAb43-treated mice exhibited intermediate IFN-γ levels in the lung on day 2 p.i. that were significantly reduced compared with IgG controls, but elevated compared with both motavizumab and D25 treatment (Fig. 2b). Lung IFN-γ levels were similar on day 4 p.i. in all M2_82_-immunized mice regardless of antibody treatment (Fig. 2b). Similar results were observed in the serum, although IFN-γ levels remained elevated on day 4 p.i. in IgG-treated controls compared with other treatment groups (Fig. 2c). These results suggest that prophylactic neutralizing antibody treatment significantly reduces IFN-γ levels in the lung and serum, resulting in protection against memory CD8 T cell-mediated morbidity and mortality.

### Memory CD8 T cell activation and IFN-γ production are significantly reduced by pre-existing neutralizing antibodies

**Fig. 3:**
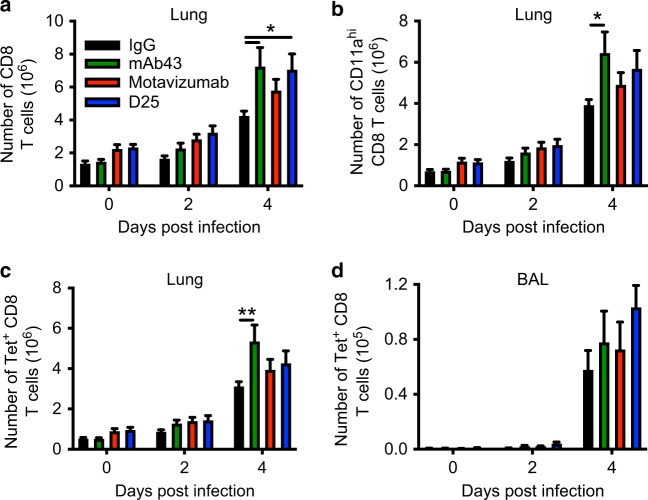
Prophylactic neutralizing antibody treatment does not impact the expansion of CD8 T cells in the lung following RSV challenge. M2_82_-immunized mice were treated with IgG, mAb43, motavizumab, or D25 antibodies i.p. and challenged with RSV i.n. 1 day later. Lungs and BAL were harvested on days 0, 2, and 4 p.i. The total numbers of (**a**) CD8 T cells, (**b**) CD11a^hi^ CD8 T cells, and (**c**) M2_82_-tetramer^+^ CD8 T cells in the lung. **d** The total numbers of M2_82_-tetramer^+^ CD8 T cells in the BAL. Data are presented as mean ± SEM of two independent experiments (*n* = 8). Groups were compared using a two-way ANOVA. *p < 0.05, **p < 0.01.

We previously showed that memory CD8 T cells were the primary cell type responsible for producing IFN-γ in M2_82_-immunized mice following RSV infection.^22^ Given that neutralizing antibody treatment in M2_82_-immunized mice substantially reduced IFN-γ levels, we next evaluated whether neutralizing antibody administration also affected the memory CD8 T cell response. We observed an increase in the total number of memory CD8 T cells in the lungs by day 4 p.i. in IgG-treated M2_82_-immunized mice (Fig. 3a). Interestingly, treatment with either mAb43 or D25 resulted in significantly increased numbers of memory CD8 T cells in the lung compared with IgG controls, while motavizumab-treated mice exhibited a trending, but not significant, elevation (Fig. 3a). Similar results were observed with both the total number of memory CD8 T cells in the lung expressing the surrogate activation marker CD11a and memory CD8 T cells specific to the M2_82_ CD8 T cell epitope (Fig. 3b, c).^29^ This is consistent with the increased PVA observed by histology, suggesting an increase in trafficking to the lung on day 4 p.i. in D25-treated mice compared with IgG controls (Fig. 1f, g). In addition, the total number of M2_82_-specific memory CD8 T cells in the BAL were similar between IgG-, mAb43-, motavizumab-, and D25-treated mice following RSV infection (Fig. 3d). These results suggest that although prophylactic neutralizing antibody treatment restricts early virus replication, it does not significantly impact the expansion of RSV-specific memory CD8 T cells in the lung and airways following RSV infection.

**Fig. 4:**
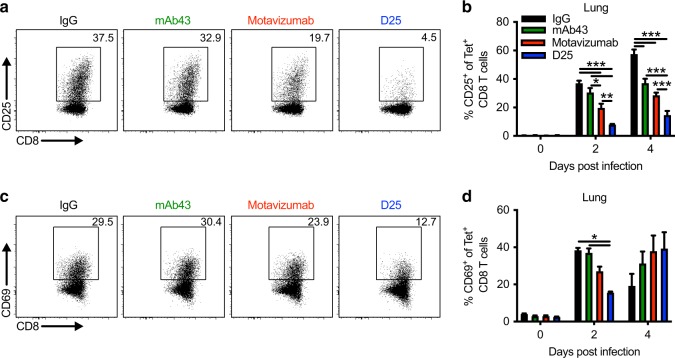
Prophylactic neutralizing antibody treatment reduces memory CD8 T cell activation in the lung following RSV challenge. M2_82_-immunized mice were treated with IgG, mAb43, motavizumab, or D25 antibodies i.p., and challenged with RSV i.n. 1 day later. Lungs were harvested on days 0, 2, and 4 p.i., and cells were gated on M2_82_-tetramer^+^ CD8 T cells. **a** Representative staining panels on day 2 p.i. and (**b**) frequency of M2_82_-tetramer^+^ CD8 T cells expressing CD25 in the lung. **c** Representative staining panels on day 2 p.i. and (**d**) frequency of M2_82_-tetramer^+^ CD8 T cells expressing CD69 in the lung. Data are presented as mean ± SEM of two independent experiments (*n* = 8). Groups were compared using a two-way ANOVA. *p < 0.05, **p < 0.01, ***p < 0.001.

We next determined the impact of neutralizing antibody treatment on the reactivation of M2_82_-specific memory CD8 T cells following RSV infection. Memory CD8 T cells rapidly upregulate activation markers including CD25 and CD69 after secondary infection.^30^ Therefore, we evaluated the expression of CD25 and CD69 on RSV-specific memory CD8 T cells in the lung as a measure of their activation status. M2_82_-specific memory CD8 T cells expressed low levels of both CD25 and CD69 in the lung prior to secondary RSV infection on day 0 p.i. (Fig. 4b, d). Control IgG and mAb43-treated M2_82_-immunized mice exhibited upregulation of CD25 expression on day 2 p.i., which was significantly elevated compared with either motavizumab- or D25-treated mice (Fig. 4a, b). IgG treatment resulted in significantly increased CD25 expression compared with mAb43, motavizumab, and D25 treatment on day 4 p.i. in the lung (Fig. 4a, b). Similar to CD25 expression, IgG and mAb43 administration resulted in an increase in CD69 expression compared with either motavizumab or D25 treatment on day 2 p.i., although the increase compared with motavizumab-treated mice did not reach statistical significance (Fig. 4c, d). Overall, these results suggest that while neutralizing antibody treatment does not impact the accumulation of M2_82_-specific memory CD8 T cells in the lung, it substantially reduces their reactivation.

**Fig. 5:**
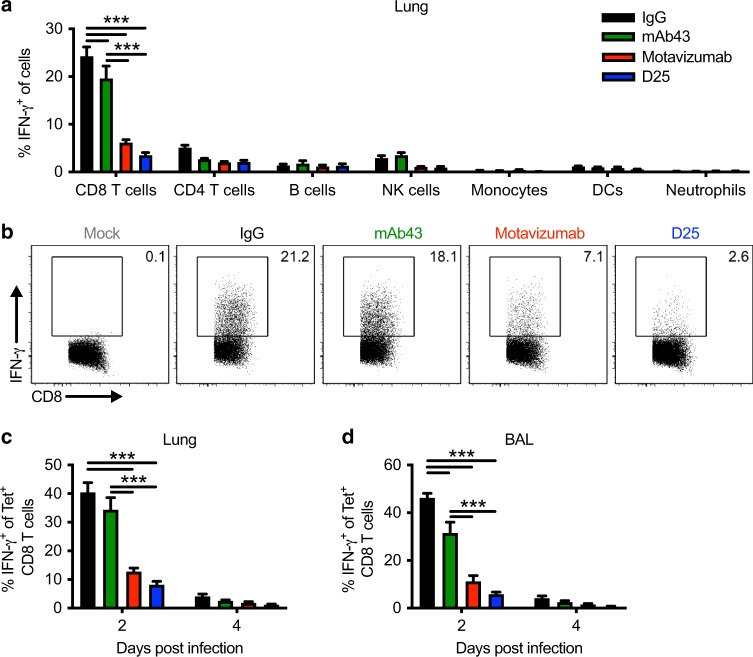
IFN-γ production by lung and airway CD8 T cells following RSV challenge is significantly reduced by prophylactic neutralizing antibody treatment. M2_82_-immunized mice were treated with IgG, mAb43, motavizumab, or D25 antibodies i.p. and challenged with RSV i.n. 1 day later. Mock controls were treated with PBS i.p. and given PBS i.n. Mice were administered 250 μg of BFA i.v. 6 h prior to tissue collection on days 2 and 4 p.i. Cells producing IFN-γ were detected by intracellular cytokine staining directly ex vivo and flow cytometry. **a** Cells were gated on the indicated cell type, and the frequency of IFN-γ producing cells are shown. **b**–**d** Cells were gated on CD8 T cells and (**b**) representative staining panels of CD8 T cells producing IFN-γ in the lung on day 2 p.i. are shown. Frequency of M2_82_-tetramer^+^ CD8 T cells producing IFN-γ in the (**c**) lung and (**d**) BAL. Data are presented as mean ± SEM of two independent experiments (*n* = 10). Groups were compared using a two-way ANOVA. ***p < 0.001.

Neutralizing antibody treatment reduced IFN-γ levels and the activation of M2_82_-specific memory CD8 T cells following RSV infection. We next evaluated whether neutralizing antibody treatment inhibited IFN-γ production by RSV-specific memory CD8 T cells. To ascertain the cellular source of IFN-γ, mice were treated with brefeldin A (BFA) in vivo to directly identify cells producing IFN-γ via intracellular cytokine staining and flow cytometry.^31^ This previously established technique allows for the identification of cytokine-producing cells in vivo without the need for ex vivo stimulation.^31^ Using this method, we previously demonstrated that IFN-γ was primarily produced by memory CD8 T cells in the lung and airways early after RSV infection in M2_82_-immunized mice.^22^ Consistent with our previous results, memory CD8 T cells produced the vast majority of IFN-γ in the lung of IgG-treated mice on day 2 p.i., with a substantially lower amount of IFN-γ made by CD4 T cells and NK cells (Fig. 5a). Memory CD8 T cells in IgG-treated mice robustly produced IFN-γ in the lung on day 2 p.i. compared with mock controls administered PBS (Fig. 5a, b). Similarly, mAb43 treatment did not significantly alter the frequency of memory CD8 T cells producing IFN-γ on day 2 p.i. (Fig. 5a, b). In contrast, administration of either motavizumab or D25 neutralizing antibodies substantially reduced the frequency of IFN-γ-producing CD8 T cells on day 2 p.i. compared with IgG-treated mice, with D25 treatment resulting in less than 5% of IFN-γ^+^ CD8 T cells in the lung (Fig. 5a, b). Similar results were observed when evaluating IFN-γ production by M2_82_-specific memory CD8 T cells in the lung on day 2 p.i. (Fig. 5c). M2_82_-specific memory CD8 T cell IFN-γ production on day 4 p.i. was decreased compared with day 2 p.i. in all treatment groups, which is consistent with our previous data (Fig. 5c).^22^ In contrast to the lung, mAb43 treatment significantly reduced the frequency of IFN-γ producing M2_82_-specific CD8 T cells in the BAL compared with IgG-treated controls (Fig. 5d). Motavizumab and D25 treatment also significantly reduced the IFN-γ production by M2_82_-specific CD8 T cells in the BAL (Fig. 5d). These results suggest that neutralizing antibody treatment significantly reduces the capacity of memory CD8 T cells in the lung and airway to produce IFN-γ following secondary RSV infection.

**Fig. 6:**
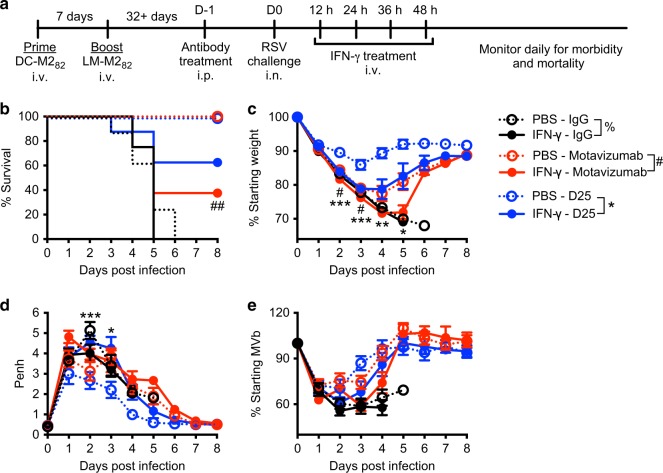
IFN-γ administration exacerbates morbidity and mortality in neutralizing antibody-treated M2_82_-immunized mice. **a** Schematic depicting immunization protocol, antibody treatment, and IFN-γ administration. M2_82_-immunized mice were treated with IgG, motavizumab, or D25 antibodies i.p., and challenged with RSV i.n. 1 day later. Mice were administered either PBS or 350 ng recombinant IFN-γ i.v. 12, 24, 36, and 48 h p.i., and monitored daily for (**b**) survival, (**c**) weight loss, (**d**) Penh, and (**e**) MVb. Data are presented as mean ± SEM of two independent experiments (*n* = 8). Groups were compared using a two-way ANOVA. ^*/#^*p* < 0.05, ^**/##^*p* *<* 0.01, ***p < 0.001.

We previously demonstrated that IFN-γ mediated the exacerbated morbidity and mortality observed following RSV infection in M2_82_-immunized mice.^22^ Here, we have shown that M2_82_-immunized mice treated prophylactically with neutralizing antibodies exhibited significantly reduced IFN-γ levels and memory CD8 T cell IFN-γ production compared with IgG-treated controls. Given these results, we evaluated whether the lack of IFN-γ in mice treated with neutralizing antibodies was the mechanism leading to their survival following RSV challenge. To demonstrate that low IFN-γ production resulted in protection against morbidity and mortality, IgG-, motavizumab-, and D25-treated M2_82_-immunized mice were administered either recombinant IFN-γ protein or PBS following RSV challenge and monitored daily for morbidity and mortality (Fig. 6a). Mice were treated systemically with four doses of recombinant IFN-γ every 12 h starting 12 h p.i. to mimic the rapid and robust induction of IFN-γ protein levels in both the lung and serum of M2_82_-immunized mice (Fig. 2b, c). As expected, IgG-treated M2_82_-immunized mice administered recombinant IFN-γ exhibited similar mortality, weight loss, and pulmonary dysfunction as PBS controls (Fig. 6b–e). Consistent with our results in Fig. 1, M2_82_-immunized mice treated with either motavizumab or D25 and administered PBS were completely protected against mortality and experienced reduced weight loss and pulmonary dysfunction compared with IgG controls (Fig. 6b–e). Remarkably, motavizumab- and D25-treated mice administered recombinant IFN-γ exhibited increased mortality following RSV challenge in contrast to their PBS control counterparts (Fig. 6b). In addition, IFN-γ administration resulted in a significant increase in weight loss and Penh, and a trending increase in MVb compared with PBS controls in both motavizumab- and D25-treated M2_82_-immunized mice (Fig. 6c–e). These results clearly demonstrate that the substantially decreased IFN-γ levels induced in neutralizing antibody-treated mice resulted in their protection against memory CD8 T cell-mediated morbidity and mortality. Overall, our data indicates that pre-existing neutralizing antibodies provide protection against memory CD8 T cell-mediated immunopathology by preventing early virus replication, which results in reduced memory CD8 T cell activation and IFN-γ production.

### Therapeutic neutralizing antibody treatment after RSV infection does not impact memory CD8 T cell-mediated immunopathology

**Fig. 7:**
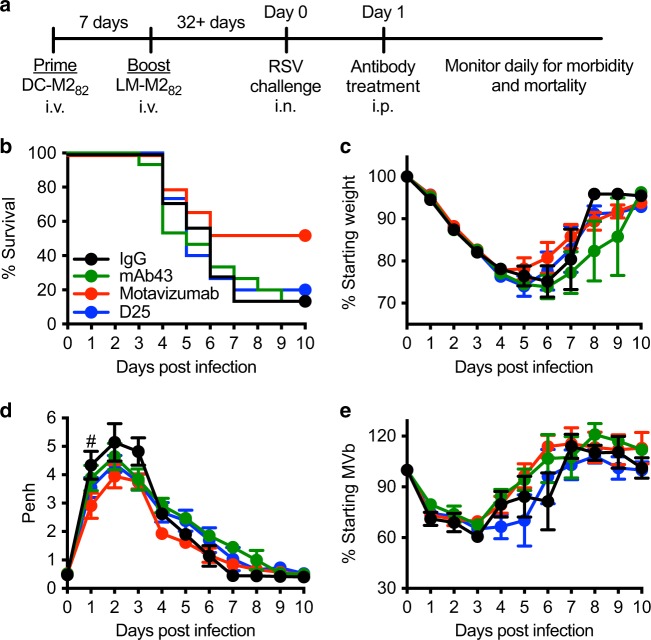
Therapeutic neutralizing antibody treatment after RSV infection does not impact memory CD8 T cell-mediated morbidity and mortality. **a** Schematic depicting immunization protocol and antibody administration. M2_82_-immunized mice were challenged with RSV and treated with IgG, mAb43, motavizumab, or D25 antibodies i.p. 1 day later. Mice were monitored daily for (**b**) survival, (**c**) weight loss, (**d**) Penh, and (**e**) MVb. Data are presented as mean ± SEM of three independent experiments (*n* = 15). Groups were compared using a two-way ANOVA. ^*/#/%^*p* < 0.05. Percent symbols represent statistical significance between IgG and mAb43 groups, pound signs represent statistical significance between IgG and motavizumab groups, and asterisks represent statistical significance between IgG and D25 groups.

Given that prophylactic neutralizing antibody treatment substantially reduced memory CD8 T cell-mediated immunopathology, we evaluated whether therapeutic administration of neutralizing antibodies after RSV challenge was also effective. M2_82_-immunized mice were challenged with RSV, treated with control IgG, mAb43, motavizumab, and D25 antibodies i.p. 1 day later, and monitored daily for morbidity and mortality (Fig. 7a). Similar to what was observed with prophylactic treatment, mAb43 administration 1 day following infection did not prevent memory CD8 T cell-mediated mortality (Fig. 7b). Surprisingly, therapeutic treatment with either motavizumab or D25 after RSV infection also did not substantially alter the survival of M2_82_-immunized mice compared with IgG-treated controls (Fig. 7b). Although motavizumab treatment reduced the percentage of mice succumbing to RSV infection to 50% mortality compared with the 90% observed in IgG-treated mice, this change was not statistically significant (*p* = 0.07) (Fig. 7b). In addition to failing to prevent mortality, therapeutic neutralizing antibody treatment also did not ameliorate memory CD8 T cell-mediated weight loss or pulmonary dysfunction, as both motavizumab- and D25-treated mice exhibited disease symptoms that were nearly identical to those treated with either control IgG or mAb43 (Fig. 7c–e). This was in stark contrast to what we observed with prophylactic neutralizing antibody treatment (Fig. 1b–e). Similarly, naive mice given neutralizing antibody treatment 1 day after a primary RSV infection exhibited significantly decreased weight loss and Penh compared with IgG-treated controls (Supplementary Fig. 2). These results indicate that therapeutic neutralizing antibody treatment is sufficient to reduce T cell-mediated disease during a primary RSV infection, but is too late to prevent the accelerated immunopathology induced following a secondary RSV challenge in mice with pre-existing memory CD8 T cell immunity. These results suggest that although pre-existing neutralizing antibodies efficiently provide protection in M2_82_-immunized mice, therapeutic treatment after RSV infection is not sufficient to overcome the rapid induction of memory CD8 T cell-mediated immunopathology.

**Fig. 8:**
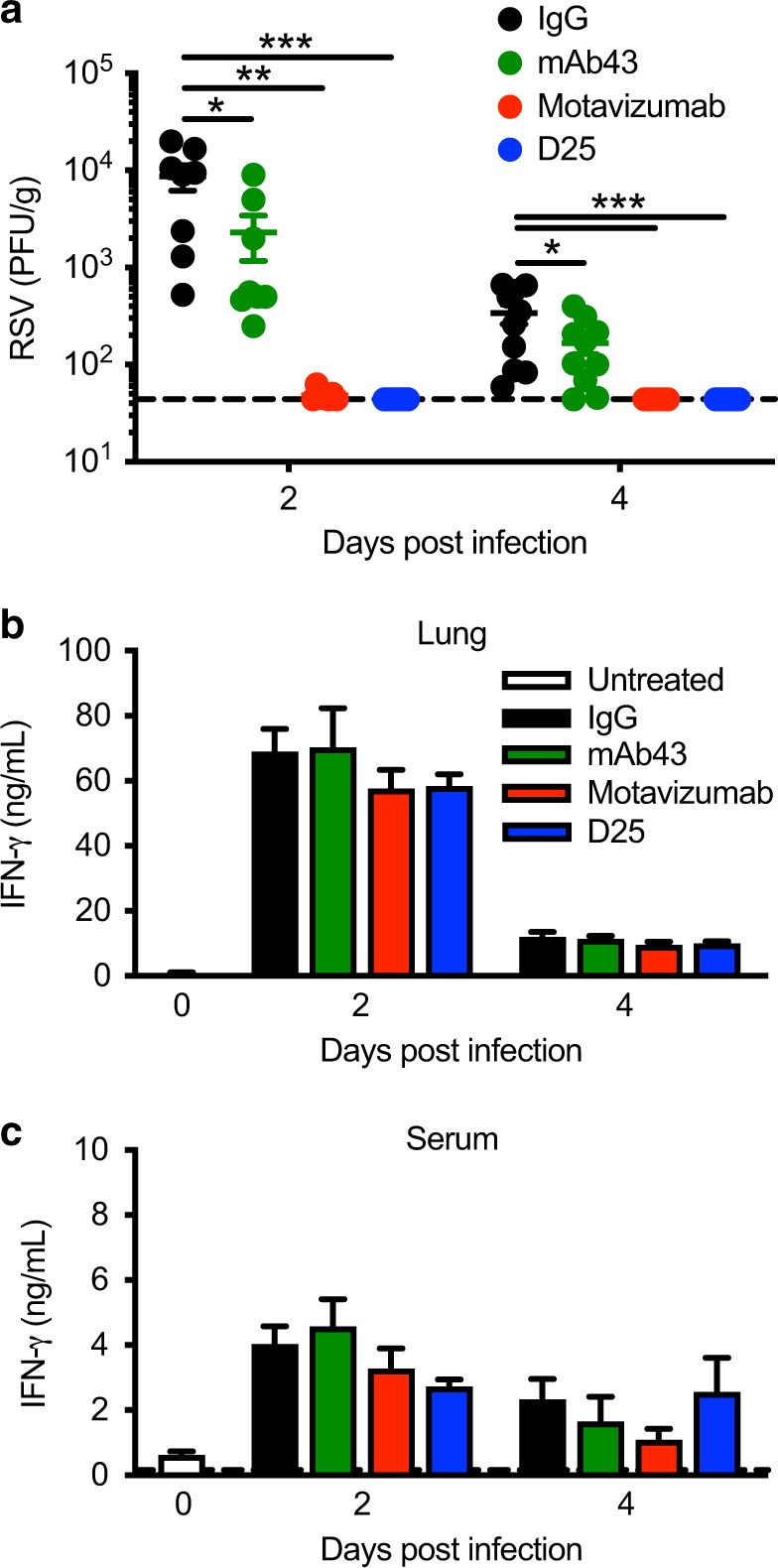
Therapeutic neutralizing antibody treatment inhibits early virus replication, but does not impact lung and serum IFN-γ levels. M2_82_-immunized mice were challenged with RSV and treated with IgG, mAb43, motavizumab, or D25 antibodies i.p. one day later. **a** Lungs were harvested on days 2 and 4 p.i., and plaque assays were performed to determine RSV titers. **b**–**c** Lungs were harvested on days 0, 2, and 4 p.i., and IFN-γ protein levels in the (**b**) lung and (**c**) serum were determined by ELISA. Data are presented as mean ± SEM of two independent experiments (*n* = 8 for day 2 and *n* = 10 for day 4). Dashed lines indicate the limit of detection of each assay. Groups were compared using a two-way ANOVA. *p < 0.05, **p < 0.01, ***p < 0.001.

To evaluate the mechanism behind the failure of therapeutic neutralizing antibody treatment to protect against CD8 T cell-mediated immunopathology, we evaluated lung viral titers and IFN-γ levels. Similar to what was observed with prophylactic antibody treatment, IgG-treated mice exhibited a high level of RSV in their lungs on day 2 p.i. that was significantly elevated compared with therapeutic treatment with mAb43, motavizumab, or D25 (Fig. 8a). In contrast, both motavizumab- and D25-treated mice displayed very low lung viral titers on day 2 p.i. and complete viral clearance was observed by day 4 p.i. (Fig. 8a). Therefore, despite being administered an entire day after RSV infection, neutralizing antibodies remained able to efficiently control early RSV replication. However, therapeutic treatment with either motavizumab or D25 did not alter lung and serum IFN-γ levels compared with IgG controls (Fig. 8b, c). Therefore, our results indicate that therapeutic neutralizing antibody treatment 1 day following RSV infection of M2_82_-immunized mice is too late to inhibit memory CD8 T cell IFN-γ production and subsequent immunopathology.

## Discussion

High-magnitude memory CD8 T cells mediate severe immunopathology following RSV infection. Here, we demonstrate that the induction of RSV-specific neutralizing antibodies prior to RSV infection provides protection against memory CD8 T cell-mediated morbidity and mortality. Prophylactic treatment with either motavizumab or D25 resulted in significantly reduced weight loss and pulmonary dysfunction as well as complete protection from mortality compared with either IgG or mAb43 treatment. Motavizumab and D25 restricted lung virus replication early following RSV challenge, which resulted in substantially reduced memory CD8 T cell activation and IFN-γ production compared with IgG controls, leading to survival of the mice. In contrast, therapeutic treatment 1 day after RSV challenge with either motavizumab or D25 did not prevent CD8 T cell-mediated morbidity, mortality, and IFN-γ levels, despite efficiently controlling early viral titers in the lung. Therefore, neutralizing antibody administration even 1 day p.i. was too late to prevent immunopathology mediated by robust memory CD8 T cell responses. These results suggest that activation of RSV-specific memory CD8 T cells occurs rapidly following RSV infection, and is not dependent on subsequent virus replication and spread. Thus, only pre-existing neutralizing antibodies provide protection against RSV-specific memory CD8 T cell-mediated immunology.

Despite binding to the RSV F protein with high affinity, mAb43 does not exhibit neutralizing activity against RSV in vitro.^26^ Consistent with these results, mAb43 administration both prior to and following RSV infection did not impact morbidity and mortality in M2_82_-immunized mice. However, our data indicate that mAb43 exerts some function in M2_82_-immunized mice following RSV infection, and is not biologically inert. mAb43-treated mice exhibited a significant reduction in lung viral titers on day 2 p.i. compared with IgG controls. Similarly, prophylactic mAb43 administration resulted in significantly reduced lung and serum IFN-γ levels on day 2 p.i. compared with IgG-treated mice. In contrast, mAb43 administration prior to RSV infection did not significantly alter CD8 T cell activation and IFN-γ production. These results suggest that mAb43 contributes to reducing the early inflammatory response in M2_82_-immunized mice independently of the CD8 T cell response. In addition to direct antibody-mediated pathogen neutralization, antibodies may contribute to viral clearance by complement activation and antibody-dependent cellular cytotoxicity (ADCC) through activity of their crystallizable fragment (Fc) domains.^32^ Antibody Fc domains can form immune complexes that activate the complement system, resulting in the recruitment of phagocytes, opsonization, and assembly of the membrane attack complex to destroy infected target cells.^33,34^ Antibody immune complex-mediated activation of complement has been previously implicated in the exacerbation of vaccine-enhanced disease following RSV challenge of formalin-inactivated RSV-immunized mice.^35^ ADCC is mediated by antibodies binding to Fc receptors expressed on NK cells, which triggers the release of perforin and granzymes to eliminate infected target cells. ADCC contributes to antibody-mediated protection against other respiratory viruses, including influenza virus.^36^ Further, human RSV-specific antibodies exhibit potent ADCC activity in vitro.^37^ Thus, mAb43 administration may be contributing to the reduction of lung viral titers through either complement activation or ADCC.

Motavizumab is the affinity-optimized, next-generation monoclonal antibody of its predecessor, palivizumab that binds to site II on both the pre-F and post-F confirmations of the RSV F protein.^23,38^ Indeed, motavizumab exhibited 10–20-fold higher in vitro neutralizing activity than palivizumab.^23,38^ In addition, motavizumab demonstrated 3–4-fold higher potency compared with palivizumab in the elimination of RSV titers in vivo in cotton rats.^23^ More recently, the monoclonal antibody D25 was identified, which binds with high affinity to site ∅ exclusively on the pre-F confirmation.^24,25^ D25 exhibited significantly increased in vitro neutralizing activity compared with either palivizumab or motavizumab.^24,25^ Further optimization of D25 yielded the highly potent, humanized monoclonal antibody, MEDI18897.^39^ MEDI18897 demonstrated in vitro neutralizing activity that was 20-fold and 150-fold more potent than motavizumab and palivizumab, respectively.^39^ Furthermore, MEDI18897 was ninefold more potent than palivizumab at inhibiting RSV replication in cotton rats.^39^ Together, these results indicate that antibodies specific exclusively to the pre-F confirmation are more effective than those capable of binding to both the pre-F and post-F forms. In addition to exerting higher neutralizing activity, our results suggest that antibodies specific to pre-F are more effective at preventing memory CD8 T cell-mediated immunopathology following RSV infection in mice. Motavizumab-treated mice exhibited nearly 20% body weight loss and notable elevations in Penh from baseline, although these parameters were significantly reduced compared with IgG controls at multiple days following infection. In contrast, D25 treatment nearly eliminated all clinical disease manifestations, as little change in weight loss and Penh values from baseline were observed. Therefore, although both motavizumab and D25 are capable of preventing memory CD8 T cell-mediated mortality following RSV infection, D25 is more efficacious in reducing clinical disease symptoms, including weight loss and pulmonary dysfunction.

Our results indicate that the severe memory CD8 T cell-mediated disease observed following RSV infection can be prevented by the presence of pre-existing neutralizing antibodies. Thus, vaccines developed to induce high-magnitude memory CD8 T cells in combination with neutralizing antibody responses may be efficacious while maintaining a suitable safety profile. Our results are consistent with those observed in human clinical trials evaluating either prophylactic or therapeutic treatment with RSV-specific neutralizing antibodies in infants. Phase III clinical trials demonstrated that prophylactic treatment with either palivizumab or motavizumab resulted in reduced RSV-associated hospitalization rates in pre-term infants.^40,41^ In contrast, therapeutic administration of either palivizumab or motavizumab to RSV-infected infants after hospitalization did not alter disease severity and the duration of hospitalization compared with placebo-treated RSV-infected infants.^42,43^ Thus, only pre-existing RSV-specific neutralizing antibodies are sufficient to reduce RSV-associated disease in humans, similar to our results reported here evaluating RSV-specific memory CD8 T cell-mediated disease in mice. Given the demonstrated ability for prophylactic administration of neutralizing antibodies to prevent RSV-associated disease in humans, it is likely that they will also be capable of mitigating potentially pathological responses mediated by human memory CD8 T cells. Overall, our findings suggest that eliciting both neutralizing antibodies and memory CD8 T cells may be an effective strategy for the development of future CD8 T cell epitope-based vaccines against RSV.

## Methods

### Mice

Female BALB/cAnNCr mice between 6–8 weeks old were purchased from the National Cancer Institute (Frederick, MD). All experimental procedures utilizing mice were approved by the University of Iowa Animal Care and Use Committee under Animal Protocol #7041999. The experiments were performed under strict accordance to the Office of Laboratory Animal Welfare guidelines and the Public Health Service Policy on Humane Care and Use of Laboratory Animals.

### Prime-boost immunizations

M2_82_-specific memory CD8 T cells were induced using a DC-LM prime-boost immunization regimen as previously described.^22^ Briefly, LPS-matured DCs were pulsed with M2_82–90_ peptide and sorted via positive selection with anti-CD11c microbeads (Miltenyi Biotec, Auburn, CA). Naive BALB/c mice were primed with 5 × 10^5^ DC-M2_82_ intravenously (i.v.) and boosted 7 days later i.v. with 5 × 10^6^ recombinant *actA*-deficient LM expressing the M2_82–90_ epitope.

### Viruses and infection

The A2 strain of RSV was a gift from Dr. Barney Graham (National Institutes of Health, Bethesda, MD) and was purified as previously described.^22^ M2_82_-immunized mice were challenged with 3.0 × 10^6^ PFU of purified RSV intranasally (i.n.) 32–40 days post boost.

### Antibody treatment

M2_82_-immunized mice were treated with 3 mg/kg of antibody diluted in PBS i.p. either 1 day before or 1 day after RSV challenge as indicated in the figure legends. Antibody doses were calculated per individual mouse according to their starting weight on either the day of antibody administration or the day of RSV challenge. RSV-specific neutralizing antibodies motavizumab and D25 and the RSV-specific non-neutralizing mAb43 were provided by MedImmune (Gaithersburg, MD). Control rat IgG was purchased from MP Biomedicals, LLC (Santa Ana, CA).

### Assessment of weight loss and pulmonary function

Mice were monitored daily following infection for weight loss. Mice with weights at or below 70% of their starting weight were euthanized. Pulmonary function was evaluated using an unrestrained whole-body plethysmograph (Data Sciences International, New Brighton, MN). Whole-body plethysmography parameters were measured as changes in respiration from baseline values prior to infection and did not utilize methacholine administration. Pressure and volume changes in the chamber caused by respiration were averaged over a 5-min period and used to calculate Penh and minute volume (MVb).

### Histology

Whole lungs were harvested on day 4 p.i. and fixed in 10% formalin (Fisher Scientific, Hampton, NH). Fixed tissues were processed and embedded in paraffin at the Comparative Pathology Laboratory (University of Iowa). Paraffin blocks were sectioned at four-micron thickness and stained for hematoxylin and eosin (H&E). Tissues were examined by a board-certified veterinary pathologist and scored in a manner masked to experimental groups following principles for reproducible scoring.^44,45^ Tissues were assigned scores for PVA and HM using a 1–4 ordinal system as previously described.^46^ PVA scoring was performed as follows: (1) within normal parameters; (2) focal solitary cells with uncommon aggregates; (3) multifocal nominal-to- moderate-sized aggregates; (4) moderate-to-high cellularity, multifocal large cellular aggregates that may be expansive into adjacent tissues. HM scoring was performed as follows: (1) absent; (2) rare, 0–33%, airspace lined by eosinophilic fibrillar material and cellular debris; (3) moderate, 33–66%; (4) extensive/coalescing fields, >66% of lung fields.

### Plaque assay

Whole lungs were harvested from mice, weighed, mechanically homogenized, and supernatants were flash-frozen in liquid nitrogen prior to storage at −80 °C. Plaque assays were performed using VERO cells (American Type Culture Collection, Manassas, VA) to determine RSV titers as previously described.^22^

### ELISA

Serum was collected and whole lungs were harvested on days 0, 2, and 4 p.i. Lungs were disrupted using a tissue homogenizer (Ultra-Turrax T25; IKA Works, Inc., Wilmington, NC), lung homogenates were centrifuged at 2000 rpm for 10 min, and supernatants were collected. Lung and serum IFN-γ levels were determined by ELISA as previously described.^47^

### Flow cytometry analysis

Lungs and BAL fluid were harvested from mice, and single-cell suspensions were generated as previously described.^48,49^ Cells were stained extracellularly with CD8 tetramers (RSV M2_82–90_; made in house), and monoclonal antibodies specific to CD90.2 (clone 53–2.1), CD4 (clone RM4–5), CD8 (clone 53–6.7), CD11a (clone M17/4), CD25 (clone PC61; BioLegend, San Diego, CA), CD69 (clone H1.2F3), CD4 (clone GK1.5), CD3 (clone 145–2C11), CD19 (clone 6D5), B220 (clone RA3–6B2), CD49b (clone DX5), NKp46 (clone 29A1.4), Ly6G (clone 1A8), Siglec F (clone E50–2440; BD Biosciences, San Jose, CA), MHC class II (clone M5/114.15.2), CD64 (clone X54–5/7.1), CD11b (clone M1/70), and CD11c (clone N418) in FACS buffer (PBS, 2% FCS, 0.02% sodium azide) for 30 min at 4 °C and fixed using 1-step Fix/Lyse Solution for 10 min at room temperature (eBioscience). For intracellular cytokine staining, cells were stained for surface markers as indicated above and subsequently stained intracellularly for IFN-γ (clone XMG1.2) in FACS buffer containing 0.5% saponin (Sigma-Aldrich, St. Louis, MO) for 30 min at 4 °C. All antibodies were purchased from eBioscience unless otherwise indicated. Samples were run on a BD LSRFortessa (BD Biosciences) and analyzed using FlowJo software (Tree Star, Inc., Ashland, OR). Cell types were phenotyped as follows: CD8 T cells (CD90.2^+^CD8^+^), CD4 T cells (CD90.2^+^CD4^+^), B cells (CD3^−^CD19^+^B220^+^), NK cells (CD3^−^CD49b^+^NKp46^+^), monocytes (Ly6G^−^Siglec F^−^SSC-A^lo^MHC class II^lo^CD64^hi^CD11b^hi^), dendritic cells (Ly6G^−^Siglec F^-^CD11c^+^MHCII^+^), and neutrophils (Siglec F^−^Ly6G^+^).

### In vivo BFA administration

CD8 T cells producing IFN-γ in vivo were detected using in vivo BFA administration as previously described.^31^ M2_82_-immunized mice were injected with 250 µg BFA (Sigma-Aldrich) in 500 µl PBS i.v. Lungs and BAL were harvested 6 h later, and cells were stained as indicated above directly ex vivo without restimulation. Mock-treated M2_82_-immunized mice were administered 100 µl PBS i.p. and 100 µl PBS i.n. in place of antibody treatment and RSV infection, respectively.

### IFN-γ treatment

Antibody-treated M2_82_-immunized mice were administered 350 ng recombinant IFN-γ (BioLegend) in 50 µl PBS i.v. 12, 24, 36, and 48 h post-RSV challenge. PBS control antibody-treated M2_82_-immunized mice were injected with 50 µl PBS i.v. 12, 24, 36, and 48 h post-RSV challenge.

### Statistical analysis

All statistical analyses are described in each figure legend and were performed using Prism software (GraphPad Software, San Diego, CA). Data were evaluated using either a one-way ANOVA or two-way ANOVA with Tukey-Kramer’s post test for more than two groups to determine a statistical significance of at least α = 0.05 as indicated in the figure legends.

## Supplementary information

Supplementary Figure Legends
