# Supplementary material for: Pre-existing neutralizing antibodies prevent CD8 T cell-mediated immunopathology following respiratory syncytial virus infection
Source: Mucosal Immunol. 2019 Dec 16;13(3):507–17. doi: 10.1038/s41385-019-0243-4 (PMC7181396; doi:10.1038/s41385-019-0243-4)
Supplement: Supplementary file 1 — Supplementary Figure Legends [file 41385_2019_243_MOESM1_ESM.pdf]

Supplemental Figure Legends

Supplemental Figure 1. Prophylactic neutralizing antibody treatment protects $M2_{82}$-immunized mice from diffuse alveolar damage.

$M2_{82}$-immunized mice were treated with IgG or D25 antibodies i.p. and challenged with RSV i.n. one day later. Lungs were harvested on day 4 p.i. from $M2_{82}$-immunized mice treated with either IgG or D25 and naive mice. Sections were stained for H&E, and representative images were captured at 20X magnification. Black arrowheads indicate PVA.

Supplemental Figure 2. Therapeutic neutralizing antibody treatment ameliorates disease following a primary RSV infection.

(a) Schematic depicting RSV infection and antibody administration. Naive mice were infected with RSV and treated with IgG, mAb43, motavizumab, or D25 antibodies i.p. one day later. Mice were monitored daily for **(b)** weight loss and **(c)** Penh. Data are presented as mean ± SEM of 2 independent experiments (n=8). Groups were compared using a two-way ANOVA. */# $p<0.05$, **/## $p<0.01$, ***/### $p<0.001$. Pound signs represent statistical significance between IgG and motavizumab groups, and asterisks represent statistical significance between IgG and D25 groups.

FIG S1

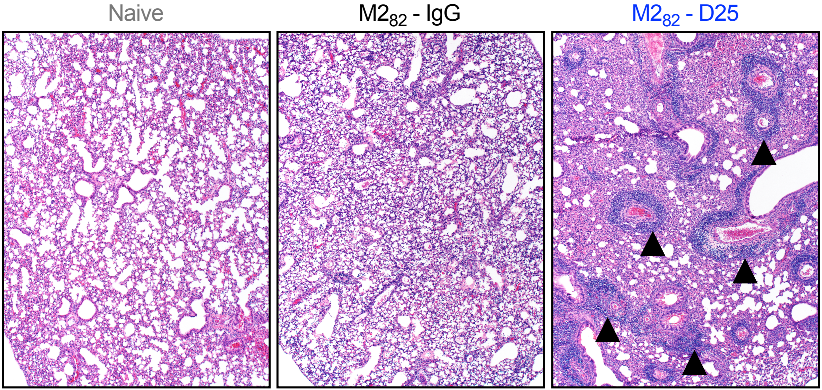

FIG S2

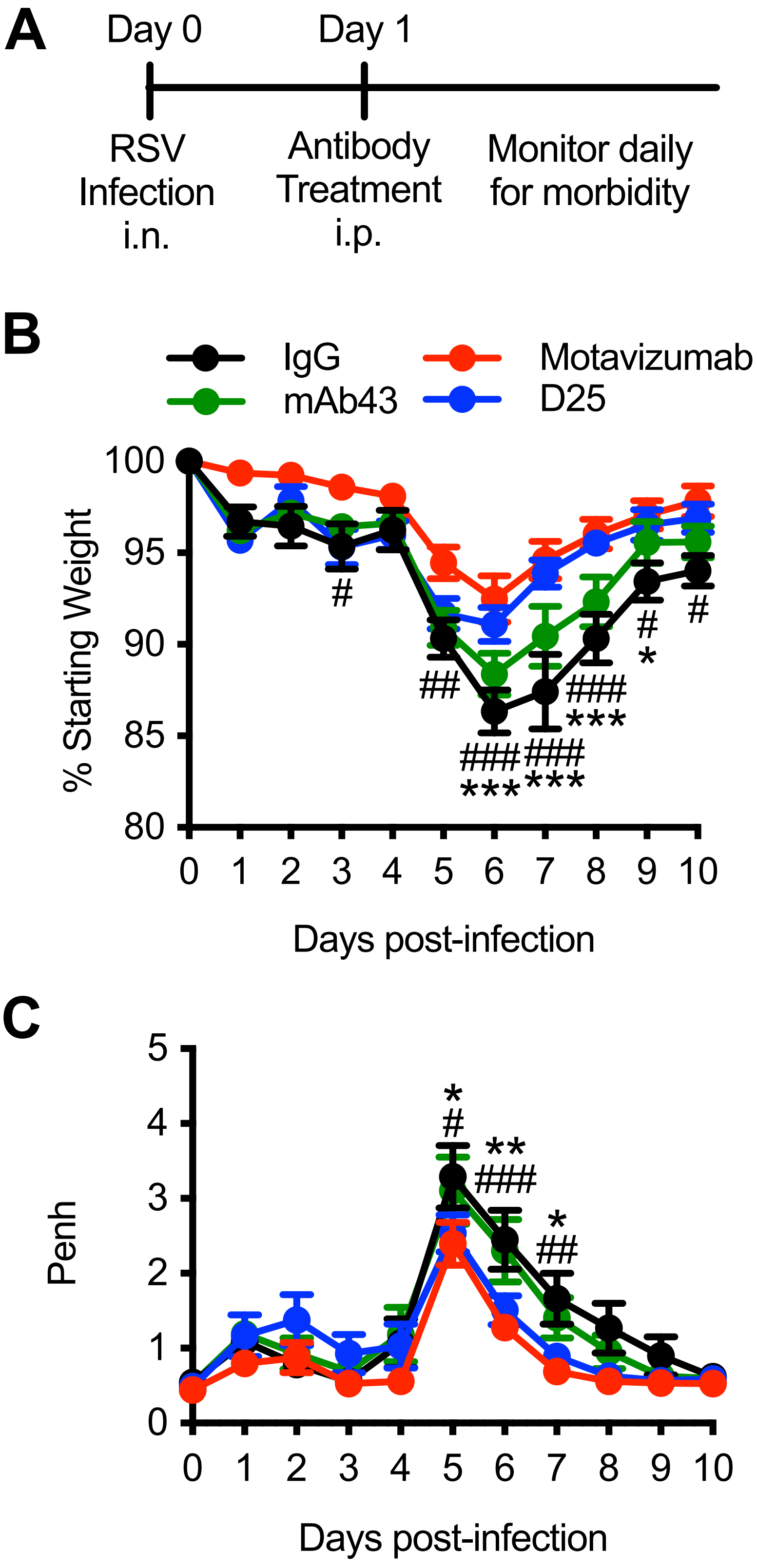
A
Day 0
Day 1
RSV Infection i.n.
Antibody Treatment i.p.
Monitor daily for morbidity
B
IgG
mAb43
Motavizumab
D25
% Starting Weight
100
95
90
85
80
#
##
###
***
###
***
###
***
#
*
#
0 1 2 3 4 5 6 7 8 9 10
Days post-infection
C
Penh
5
4
3
2
1
0
*
#
**
###
*
##
0 1 2 3 4 5 6 7 8 9 10
Days post-infection
